# Public Support for Smoke-Free Air Strategies Among Smokers and Nonsmokers, New York City, 2010–2012

**DOI:** 10.5888/pcd11.130263

**Published:** 2014-01-30

**Authors:** Elizabeth Needham Waddell, Shannon M. Farley, Jenna Mandel-Ricci, Susan M. Kansagra

**Affiliations:** Author Affiliations: Elizabeth Needham Waddell, New York City Department of Health and Mental Hygiene and Waddell Research, Portland, Oregon; Jenna Mandel-Ricci, Susan M. Kansagra, Bureau of Chronic Disease Prevention and Tobacco Control, New York City Department of Health and Mental Hygiene, New York, New York.

## Abstract

**Introduction:**

From 2010 through 2012, the New York City Department of Health and Mental Hygiene engaged in multiple smoke-free-air activities in collaboration with community, institution, and government partners. These included implementing a law prohibiting smoking in all parks and beaches as well as working to increase compliance with existing Smoke-free Air Act provisions.

**Methods:**

We investigated trends in awareness of existing smoke-free rules publicized with new signage and public support for new smoke-free air strategies by using 3 waves of survey data from population-based samples of smoking and nonsmoking adults in New York City (2010–2012). Analyses adjusted for the influence of sociodemographic characteristics.

**Results:**

Among both smokers and nonsmokers, we observed increased awareness of smoke-free regulations in outdoor areas around hospital entrances and grounds and in lines in outdoor waiting areas for buses and taxis. Regardless of smoking status, women, racial/ethnic minorities, and adults aged 25 to 44 years were more likely than men, non-Hispanic whites, and adults aged 65 years or older to support smoke-free air strategies.

**Conclusion:**

New signage was successful in increasing population-wide awareness of rules. Our analysis of the association between demographic characteristics and support for tobacco control over time provide important contextual information for community education efforts on secondhand smoke and smoke-free air strategies.

## Introduction

Data from population surveys have demonstrated that support for tobacco control has increased substantially over the past 3 decades as smoking rates have declined and public health awareness and the number of antismoking policies have grown (1–6). After a cigarette sales tax increase and passage of a comprehensive indoor air law in New York City (NYC) called the Smoke-Free Air Act (SFAA) in 2002, smoking prevalence declined from 21.5% in 2002 to 19.2% in 2003, and continued the decline to 14.8% in 2011 (7,8). Nearly half of New Yorkers surveyed after passage of smoke-free air legislation reported less exposure to environmental tobacco smoke (7). Since passage of the SFAA of 2002, additional interventions and policies have expanded smoke-free air in NYC. These include legislative expansion of the SFAA to include hospital grounds, public parks, and beaches; voluntary policy changes by institutions; and educational activities to increase awareness of and compliance with smoke-free air rules. However, public awareness and support for smoke-free air strategies has not been measured at the citywide level. Between March 2010 and March 2012, the NYC Department of Health and Mental Hygiene (DOHMH) implemented multiple activities to promote smoke-free air. These activities were made possible through the Communities Putting Prevention to Work (CPPW) grant from the Centers for Disease Control and Prevention, which enabled NYC to develop partnerships with community institutions and implement community-level educational initiatives to promote smoke-free air strategies aimed at decreasing tobacco use and secondhand smoke exposure (9). Activities were as follows:

- DOHMH created institutional partnerships with NYC hospitals to improve compliance with existing smoke-free air laws for hospital grounds, including outdoor areas, and to improve cessation services for employees, patients, and visitors. The first hospital partnership was formed in April 2011, and 6 more hospitals joined the program by November 2011. Updating all hospital campus signage to designate tobacco-free areas was a first step in the partnership, including signage in multiple languages to meet community needs.
- DOHMH partnered with the Port Authority of New York and New Jersey to post signs prohibiting smoking in outdoor queues in April of 2011, protecting the millions of travelers who pass through its 2 airports and 2 bus terminals each year. A total of 71 signs were installed at all taxi lines at LaGuardia Airport, John F. Kennedy International Airport, Port Authority Bus Terminal, and George Washington Bridge Bus Terminal.
- DOHMH developed a citywide print and television media campaign that aired from May 26, 2011, to June 12, 2011, to increase public awareness of the 2010 legislation that prohibited smoking in all of NYC’s 1,700 public parks and 14 miles of beaches, as well as all pedestrian plazas. The media campaign included TV ads in English and Spanish, as well as subway and bus ads. Additionally, “no smoking” signage was posted at entrances to all parks and beaches.

We used data from the NYC Tobacco Behavior and Public Opinion Survey (TBPOS), a cross-sectional survey administered 3 times over 2 years, to assess New Yorkers’ exposure to environmental tobacco smoke and to evaluate changes in awareness of and support for smoke-free air rules as a result of these efforts. We also determined sociodemographic characteristics associated with support for smoke-free air rules, which could inform educational efforts on smoke-free air strategies in other jurisdictions. Similar surveys have assessed public opinion around smoke-free environments at the state and national levels (3,5,10–14). We examined changes in local support for tobacco control over a 2-year period.

## Methods

DOHMH conducted 3 waves of the TBPOS (wave 1 in August 2010, wave 2 in April 2011, and wave 3 in February 2012). The TBPOS was a citywide landline and cellular-phone cross-sectional survey, which was implemented by a survey research firm and approved by the DOHMH’s institutional review board. Participants were identified through landline (90% of sample) and cell-phone (10% of sample) numbers. Participants in the landline component did not receive any compensation. Participants in the cell-phone sample were mailed a $4.50 MetroCard (public transit pass) to cover the expense of the call.

Interviewers called each telephone number up to 15 times, with a goal of enrolling 1,440 participants over a 1-month period, including 720 current smokers and 720 nonsmokers. Smoking status was obtained at the time of the interview as part of eligibility screening.

Surveys were administered in English and Spanish. Current smoking was determined by 2 questions, “Have you smoked at least 100 cigarettes in your entire life?” and “Do you now smoke cigarettes every day, some days, or not at all?” To assess perceptions of secondhand smoke exposure in the areas targeted by CPPW initiatives, participants were asked if they thought secondhand smoke was a problem 1) in outdoor public places, within a few feet of them; 2) in the areas around hospital entrances or on hospital grounds; and 3) in lines in outdoor waiting areas for a bus or taxi at NYC airports or Port Authority and George Washington Bridge bus terminals. To assess knowledge of existing no-smoking regulations, participants were asked a series of questions focused on 1) hospital entrances or on hospital grounds and 2) outdoor waiting areas for a bus or taxi. For each location, participants indicated whether they thought smoking was allowed or prohibited and whether they had noticed in the last 3 months any signs prohibiting smoking. To assess public opinion regarding smoke-free air strategies, participants were asked if they favored or opposed prohibiting smoking 1) in all public parks and 2) on all public beaches. Additional survey questions assessed sex (male/female), age in years, race/ethnicity (non-Hispanic black, non-Hispanic white, Hispanic/Latino, Asian-American/Other), language spoken most often at home, and education level. Participants were also specifically asked if they considered themselves “a Hispanic, Latino, or Spanish-speaking American.”

During wave 1 (pre-intervention), 685 smokers and 762 nonsmokers were surveyed; in wave 2 (pre-intervention), 715 smokers and 721 nonsmokers were surveyed; and in wave 3 (post-intervention), 718 smokers and 727 nonsmokers were surveyed. We calculated response rates and cooperation rates using the American Association for Public Opinion Research’s third definition, which does not consider partial interviews complete (15). To assess the overall representativeness of the TBPOS, we compared the demographic composition of the weighted data set (all years combined) to the 2011 Community Health Survey (CHS) population (8). We used SAS 9.2 (SAS Institute Inc, Cary, North Carolina) survey procedures to estimate weighted percentages and adjust for clustering by survey wave. Combined smoker and nonsmoker estimates were obtained by weighting observations to match smoking prevalence data that were available for NYC at the time of the survey (wave 1, 15.8%; waves 2 and 3, 14.0%) (8). Because smoking behavior was our key variable of interest, we weighted combined survey data to smoking prevalence only. We did not post-stratify the data based on demographic characteristics or telephone type.

We assessed postintervention changes using a set of orthogonal contrasts. The first contrast (wave 1 = .5, wave 2 = .5, wave 3 = −1) tests if the wave 3 (February 2012) outcome differed from waves 1 (August 2010) and 2 (April 2011). The second contrast (wave 1 = 1; wave 2 = −1; wave 3 = 0) tests if waves 1 and 2 differ; including the second contrast helps account for any variance between these waves. Each outcome was regressed on the 2 contrasts simultaneously in logistic regression models. Change from pre- to postintervention was indicated by a statistically significant (*P* < .05) coefficient on the first contrast. To adjust for potential demographic differences across waves, we ran additional regression models that controlled for age group, education, race/ethnicity, sex, and language spoken most often at home (English vs Spanish/other). We assessed change for smokers and nonsmokers separately and combined.

To test for demographic differences in public opinion on smoke-free air strategies, controlling for smoking status, we fit multivariable logistic regression models for support for smoke-free parks and beaches. We initially included main effects only and removed variables one at a time when the *P* value was less than .10. We then added all possible 2-way interaction terms between smoking and significant demographic characteristics and interactions when the *P* value was less than .10.

## Results

Response rates in wave 1 were 21.3% for landlines and 8.6% for cellular phones, with cooperation rates of 50.6% for landlines and 10.1% for cellular phones; in wave 2 the response rates were 23.7% for landlines and 3.3% for cellular phones, with cooperation rates of 59.0% for landlines and 3.9% for cellular phones; and in wave 3 the response rates were 21.1% for landlines and 2.9% for cellular phones, with cooperation rates of 54.1% for landlines and 3.4% for cellular phones.

Compared with the CHS population, TBPOS participants were more likely to be female (63%; 95% CI, 61–65 vs 54%; 95% CI, 52–56), white/non-Hispanic (55%; 95% CI, 53–57 vs 35%; 95% CI, 34–37), and college graduates (53%; 95% CI, 51–55 vs 32%; 95% CI, 30–34) and less likely to be in the group aged 25 to 44 years (27%; 95% CI, 26–29 vs 40%; 95% CI, 38–42). These patterns were similar among smokers and nonsmokers. At wave 3 of the TBPOS, the majority of respondents overall reported that secondhand smoke was a problem in outdoor public places in NYC within a few feet of them (59%), in the areas around hospital entrances or on hospital grounds (76%), and in lines in outdoor waiting areas for a bus or taxi at NYC airports or Port Authority and George Washington Bridge bus terminals (65%). Among smokers and nonsmokers combined, there was no significant difference in these areas between pre- and postintervention surveys [Table 1]

**Table 1:** Trends in Public Awareness and Perceptions of Secondhand Smoke and Smoke-Free Air Strategies, Tobacco Behavior and Public Opinion Survey, New York City, 2010, 2011, 2012

Outcome	Weighted Percentage			*P* Value, Pre vs Post^a^	Adjusted *P* Value, Pre vs Post^a^^,^ b
	Pre Wave 1 2010	Pre Wave 2 2011	Post Wave 3 2012		
**Secondhand smoke**					
Secondhand smoke is a problem in outdoor public places in New York City within a few feet of me	60	62	59	.25	.67
Secondhand smoke is a problem in the areas around hospital entrances or on hospital grounds in New York City	72	75	76	.21	.22
Secondhand smoke is a problem in lines in outdoor waiting areas for a bus or taxi at New York City airports or Port Authority and George Washington Bridge bus terminals?	64	68	65	.64	.87
**Existing smoke-free air regulations**					
Smoking is prohibited in the areas around hospital entrances or on hospital grounds in New York City	48	53	55	.04	.07
Noticed signs the last 3 months prohibiting smoking in the areas around hospital entrances or on hospital grounds in New York City	39	39	41	.31	.37
Smoking is prohibited in lines in outdoor waiting areas for a bus or taxi at New York City airports or Port Authority and George Washington Bridge bus terminals	26	27	32	<.001	<.001
Noticed signs the last 3 months prohibiting smoking in lines in outdoor waiting areas for a bus or taxi at New York City airports or Port Authority and George Washington Bridge bus terminals	15	11	15	.04	.007
**Support for smoke-free air strategies**					
Favor prohibiting smoking in all public parks	52	46	47	.46	.47
Favor prohibiting smoking on all public beaches	48	44	50	.06	.23

a Logistic regression model contrasting weighted average of 2010/2011 (pre) values with 2012 (post) values. Model controls for change from 2010 to 2011.

b Model controls for sex, age group, race/ethnicity, language usually spoken at home, and education.

More than half (55%) of respondents at wave 3 were aware that smoking is prohibited the areas around hospital entrances and on hospital grounds in New York City, which was a significant improvement (*P* = .04) over wave 1 (48%) and wave 2 (53%). This improvement was no longer significant (*P* = .07) after controlling for demographic characteristics. Fewer than half of respondents overall (41%) had noticed signs that prohibited smoking in areas around hospital entrances or on hospital grounds by wave 3, similar to waves 1 and 2.

A significantly (*P* < .001) greater proportion of respondents at wave 3 (32%) were aware that smoking is prohibited in bus and taxi lines at NYC airports and bus terminals than at waves 1 and 2 (26% and 27%, respectively). Only 15% of respondents overall had noticed signs prohibiting smoking in these areas by wave 3, which represented a significant (*P* = .04) increase from waves 1 and 2 (15% and 11%, respectively), even after adjusting for demographics (*P* = .007).

Among the combined population of smokers and nonsmokers, support for prohibiting smoking in all public parks (47% at wave 3) and on all public beaches (50% at wave 3) was consistent over the 3 survey waves (Table 2).

**Table 2:** Trends in Public Awareness and Perceptions of Secondhand Smoke and Smoke-Free Air Strategies by Smoking Status, Tobacco Behavior and Public Opinion Survey, New York City, 2010, 2011, 2012

Outcome	Current Smokers					Nonsmokers				
	Unweighted Percentage			*P* Value, Pre vs Post^a^	Adjusted *P* Value, Pre vs Post^a^^,^ b	Unweighted Percentage			*P* Value, Pre vs Post^a^	Adjusted *P* value, Pre vs Post^a^^,^ b
	Pre Wave 1, 2010	Pre Wave 2, 2011	Post Wave 3, 2012			Pre Wave 1, 2010	Pre Wave 2, 2011	Post Wave 3, 2012		
**Secondhand smoke**										
Secondhand smoke is a problem in outdoor public places in New York City within a few feet of me	30	33	24	<.001	<.001	66	67	65	.42	.82
Secondhand smoke is a problem in areas around hospital entrances or on hospital grounds in New York City	54	63	55	.12	.30	76	77	79	.14	.20
Secondhand smoke is a problem in lines in outdoor waiting areas for a bus or taxi at New York City airports or Port Authority and George Washington Bridge bus terminals	36	39	37	.99	.84	70	73	70	.52	.88
**Existing smoke-free air regulations**										
Smoking is prohibited in the areas around hospital entrances or on hospital grounds in New York City	60	61	67	.006	.005	45	52	53	.09	.13
Noticed signs the last 3 months prohibiting smoking in the areas around hospital entrances or on hospital grounds in New York City	59	55	62	.02	.007	36	37	38	.46	.58
Smoking is prohibited in lines in outdoor waiting areas for a bus or taxi at New York City airports or Port Authority and George Washington Bridge bus terminals	31	26	38	<.001	<.001	25	27	32	.005	<.001
Noticed signs the last 3 months prohibiting smoking in lines in outdoor waiting areas for a bus or taxi at New York City airports or Port Authority and George Washington Bridge bus terminals	20	15	25	<.001	<.001	14	10	14	.18	.06
**Support for smoke-free air strategies**										
Favor prohibiting smoking in all public parks	30	34	33	.69	.52	52	58	56	.56	.61
Favor prohibiting smoking on all public beaches	30	33	33	.65	.80	56	59	53	.03	.17

a Logistic regression model contrasting weighted average of 2010/2011 (pre) values with 2012 (post) values. Model controls for change from 2010 to 2011.

b Model controls for sex, age group, race/ethnicity, language usually spoken at home, and education.

Between pre- (30% and 33%, respectively) and post- (24%) waves, the proportion of smokers reporting that exposure to nearby secondhand smoke in outdoor public spaces was a problem declined significantly (*P* < .001), even after adjusting for demographics (*P* = .001).

At wave 3, 67% of smokers were aware that smoking was prohibited on hospital grounds compared with 60% at wave 1 and 61% at wave 2 (*P* = .006); the improvement remained significant after controlling for demographics (*P* = .005). Similarly, 62% of smokers had noticed signs prohibiting smoking around hospitals at wave 3, compared with 59% at wave 1 and 55% at wave 2 (*P* = .02; *P* = .007 after controlling for demographics). Awareness of no-smoking regulations in outdoor taxi and bus lines also increased among smokers (31% in wave 1 and 26% in wave 2 vs 38% in wave 3, *P* < .001 after controlling for demographics), as did smokers’ awareness of signs prohibiting smoking in outdoor lines for taxis and buses (20% in wave 1 and 15% in wave 2 vs 25% in wave 3, *P* < .001 after controlling for demographics).

Among nonsmokers, there were no significant trends in reports of secondhand smoke across survey waves and no significant changes in awareness of no-smoking rules around hospitals. Awareness that smoking is prohibited in outdoor lines for taxis and buses improved somewhat (*P* = .005, *P* < .001 after controlling for demographics) among nonsmokers between waves 1 and 2 (25% and 27%, respectively) and wave 3 (32%).

Although public support for smoke-free parks and beaches was stable among smokers, there was a slight decline in support for smoke-free beaches among nonsmokers (*P* = .03), with 56% and 59% in favor at waves 1 and 2, respectively, compared with 53% at wave 3. This trend was not statistically significant (*P* = .17) after controlling for demographics.

Table 3 displays demographic variation in support for smoke-free parks and smoke-free beaches among the overall survey population, all years combined.

**Table 3:** Association Between Public Support for Tobacco Control Strategies, Current Smoking Behavior, and Demographic Characteristics, Tobacco Behavior and Public Opinion Survey, New York City, Years 2010, 2011, 2012, Combined

Demographics	Favor Smoke-Free Parks			Favor Smoke-Free Beaches		
	Weighted Prevalence (95% CI)	Multivariable regression		Weighted Prevalence (95% CI)	Multivariable regression	
		Adjusted OR (95% CI)	Adjusted *P* Value, χ^2^		Adjusted OR (95% CI)	Adjusted *P* Value, χ^2^
**Smoking**						
Current smoker	32 (30–34)	0.39 (0.34-.44)	<.001	32 (30–34)	0.36 (.31-.41)	<.001
Nonsmoker	55 (53–57)	1 [Reference]		56 (54–58)	1 [Reference]	
**Sex**						
Female	56 (53–58)	1.45 (1.23–1.70)	<.001	56 (54–58)	1.43 (1.22–1.69)	<.001
Male	46 (43–48)	1 [Reference]		47 (44–50)	1 [Reference]	
**Age group, y**						
18–24	61 (54–68)	1.40 (0.98–2.02)	.03	57 (50–64)	1.17 (0.82–1.67)	.048
25–44	57 (54–61)	1.30 (1.04–1.62)		58 (55–62)	1.33 (1.07–1.66)	
45–64	49 (46–52)	1.02 (0.83–1.25)		50 (47–53)	1.05 (0.86–1.29)	
≥65	49 (45–52)	1 [Reference]		50 (47–54)	1 [Reference]	
**Race/ethnicity**						
Non-Hispanic black	54 (50–58)	1.27 (1.04–1.56)	<.001	50 (46–54)	0.94 (0.77–1.16)	<.002
Hispanic/Latino	62 (58–67)	1.59 (1.24–2.04)		61 (57–66)	1.53 (1.22–1.92)	
Asian/other	61 (55–67)	1.59 (1.17–2.15)		60 (54–66)	1.43 (1.07–1.92)	
Non-Hispanic white	47 (44–49)	1 [Reference]		50 (48–53)	1 [Reference]	
**Language spoken most often at home**						
Other than English	65 (60–69)	1.47 (1.13–1.91)	.004	62 (57–67)	–a	–a
English	50 (48–52)	1 [Reference]		51 (49–53)		
**Education**						
Less than high school	56 (49–62)	–a	–a	52 (45–59)	–a	–a
High school graduate/some college	52 (49–55)			50 (47–53)		
College graduate or more	51 (48–53)			54 (52–57)		

Abbreviations: CI, confidence interval; OR, odds ratio.

a We initially included smoking status, sex, age group, race/ethnicity, language spoken at home, and education in the logistic regression models. We removed terms with *P* values greater than .10 individually. We found no significant interactions between smoking status and demographic characteristics in the final models.

About one third of smokers favored smoke-free parks and beaches compared with more than half of nonsmokers (Table 3). Women were more likely than men (56% vs 46% for parks and 56% vs 47% for beaches) to favor both strategies. By age group, we observed that participants aged 25 to 44 years were more likely than those 65 years and older to favor smoke-free parks (57% vs 49%) and beaches (58% vs 50%).

More blacks (54%), Hispanics/Latinos (62%), and Asians/other (race/ethnicities) (61%) favored smoke-free parks compared with non-Hispanic whites (47%). Similarly, more Hispanics (61%) and Asians/other (60%) favored smoke-free beaches than whites (50%). Finally, participants whose principal language at home was other than English were more likely than usual English speakers to support smoke-free parks (65% vs 50%) and beaches (62% vs 51%). With 1 exception, all of these differences persisted in the multivariable regression models that simultaneously adjusted for smoking status and demographic characteristics. Education fell out of both stepwise models, and language usually spoken at home was not significantly associated with support for smoke-free beaches. There were no significant interactions observed between smoking behavior and sex, age group, race/ethnicity, or language.

## Discussion

We used trend data from the 2010–2012 TBPOS to assess the impact of public education activities on smoke-free air rules on public perception of secondhand smoke exposure, awareness of existing smoke-free air strategies, and support for smoke-free strategies that have been implemented in NYC. Among both smokers and nonsmokers, there were increases in awareness of smoke-free rules in outdoor areas around hospital entrances and grounds and in outdoor bus and taxi lines. Support among nonsmokers for new smoke-free air strategies was consistently high and stable across waves.

Adult New Yorkers’ survey responses were generally consistent with prior research that found that members of traditionally underrepresented groups (eg, women, minorities, immigrants, working poor) may be more likely to support smoke-free air strategies than their counterparts (16–20). Contrary to prior research results, younger adults in NYC displayed more support for prohibiting smoking in all public parks and on beaches than those aged 65 years or older. We posit that younger New Yorkers may be more accustomed to smoke-free air regulations, given changes in NYC since 2002.

The survey’s low response rate is its greatest limitation with regard to generalizability to NYC adults overall. NYC TBPOS response rates were somewhat lower than the New York State response to the 2009–2010 National Adult Tobacco Survey, which was 38.9% for landlines and 20.0% for cellular phones (21). Response rates for the NYC TBPOS were closer to the 2011 NYC CHS landline sample (23.7% vs 33.2%, respectively), but far lower than the cellular phone sample (3.3% vs 44.2%, respectively) (22). Future TBPOS surveys will need to improve recruitment and retention of cellular phone participants to better represent younger New Yorkers and racial/ethnic minorities.

The survey’s low cellular phone response rate likely contributed to the underrepresentation of minority respondents and respondents in the group aged 25 to 44 years. While our trend analyses controlled for demographic characteristics, our annual estimates may be skewed. Our analysis of the demographic associations with support from smoke-free air strategies suggested that women are far more likely than men to support these policies, potentially inflating the estimates. However this effect was likely counteracted by the overrepresentation of whites and underrepresentation of adults in the 25 to 44 years age group.

Our analysis does not account for variation in exposure to interventions or for variation in public opinion among smokers that could be due to current or past quit attempts or desire to quit smoking (10). Nevertheless, our observations of change in attitudes toward and awareness of smoke-free air efforts in NYC provide evidence of normative change in a climate of increased support for tobacco control.

These limitations do not affect our findings regarding the associations between demographic characteristics and support for smoke-free air strategies. The demographic variation that we observed provides important contextual information for community education efforts on secondhand smoke and smoke-free air strategies. We documented a pattern of increased support for smoke-free air regulations among women, racial/ethnic minorities, immigrants, and smokers with lower levels of education. These populations may, in fact, be the most vulnerable to secondhand smoke.

Furthermore, our results show that local efforts to increase signage and educate the public were successful in increasing population-wide knowledge about smoke-free air rules in particular areas, especially among smokers. As of 2013 over 3,000 hospitals, health care systems, or clinics in the United States had adopted smoke-free grounds rules (23), and over 600 airports were smoke-free (24). Appropriate signage and education on these rules is important for increasing compliance and access to smoke-free air. Also, over 800 jurisdictions had smoke-free parks, and over 170 jurisdictions had smoke-free beach rules across the country (25,26). Although we did not see an overall increase in support for smoke-free park and beach rules, the educational campaigns may have increased compliance and awareness in these areas as well. As jurisdictions examine existing and new smoke-free-air rules, our results provide information on ways to increase awareness of these rules.
