# Erratum, Vol. 11, January 30 Release

**DOI:** 10.5888/pcd11.130263e

**Published:** 2014-03-06

In the article, “Public Support for Smoke-Free Air Strategies Among Smokers and Nonsmokers, New York City, 2010–2012,” the authors inadvertently listed the wrong grant number in the Acknowledgments section. The correct grant number is Cooperative Agreement number 1U58DP002419–01 from the Centers for Disease Control and Prevention – Communities Putting Prevention to Work. The correction was made to our website on March 6, 2014, and appears online at http://www.cdc.gov/pcd/issues/2014/13_0263.htm. We regret any confusion or inconvenience this error may have caused.

